# The ‘active life’ of Hsp90 complexes^☆^

**DOI:** 10.1016/j.bbamcr.2011.07.020

**Published:** 2012-03

**Authors:** Chrisostomos Prodromou

**Affiliations:** Genome Damage and Stability Centre, Science Park Rd, Falmer, Brighton, East Sussex BN1 9RQ, UK

**Keywords:** Hsp90, Heat shock protein 90, Chaperone, Cochaperone, Stress response, ATPase activity

## Abstract

Hsp90 forms a variety of complexes differing both in clientele and co-chaperones. Central to the role of co-chaperones in the formation of Hsp90 complexes is the delivery of client proteins and the regulation of the ATPase activity of Hsp90. Determining the mechanisms by which co-chaperones regulate Hsp90 is essential in understanding the assembly of these complexes and the activation and maturation of Hsp90's clientele. Mechanistically, co-chaperones alter the kinetics of the ATP-coupled conformational changes of Hsp90. The structural changes leading to the formation of a catalytically active unit involve all regions of the Hsp90 dimer. Their complexity has allowed different orthologues of Hsp90 to evolve kinetically in slightly different ways. The interaction of the cytosolic Hsp90 with a variety of co-chaperones lends itself to a complex set of different regulatory mechanisms that modulate Hsp90's conformation and ATPase activity. It also appears that the conformational switches of Hsp90 are not necessarily coupled under all circumstances. Here, I described different co-chaperone complexes and then discuss in detail the mechanisms and role that specific co-chaperones play in this. I will also discuss emerging evidence that post-translational modifications also affect the ATPase activity of Hsp90, and thus complex formation. Finally, I will present evidence showing how Hsp90's active site, although being highly conserved, can be altered to show resistance to drug binding, but still maintain ATP binding and ATPase activity. Such changes are therefore unlikely to significantly alter Hsp90's interactions with client proteins and co-chaperones. This article is part of a Special Issue entitled: Heat Shock Protein 90 (HSP90)

## Highlights

► The regulatory role of co-chaperones in the formation of Hsp90 complexes is reviewed. ► I summarize the role of Hsp90 co-chaperones that regulate the ATPase cycle of Hsp90. ► The role of post-translational modification on the Hsp90 chaperone cycle. ► Hsp90s active site although highly conserved can mutate without loss of function.

## 1 Introduction

Hsp90 is an essential molecular chaperone required for the maintenance, activation or maturation of specific client proteins. Many of these clients are some of the most important proteins involved in signal transduction pathways. They include proteins such as BRaf, ErbB2, Cdk4, and steroid hormone receptors as well as structural proteins such as actin and tubulin (see http://www.picard.ch/Downloads/Hsp90interactors.pdf). The versatility of the Hsp90 system has also enabled viral proteins in many instances to recruit Hsp90 as a chaperone for their own needs [1]. Because Hsp90 is responsible for the activation of such a structurally diverse range of proteins it has evolved to form a variety of complexes each with specific co-chaperones that regulate its ATPase coupled conformational changes.

**Fig. 1:**
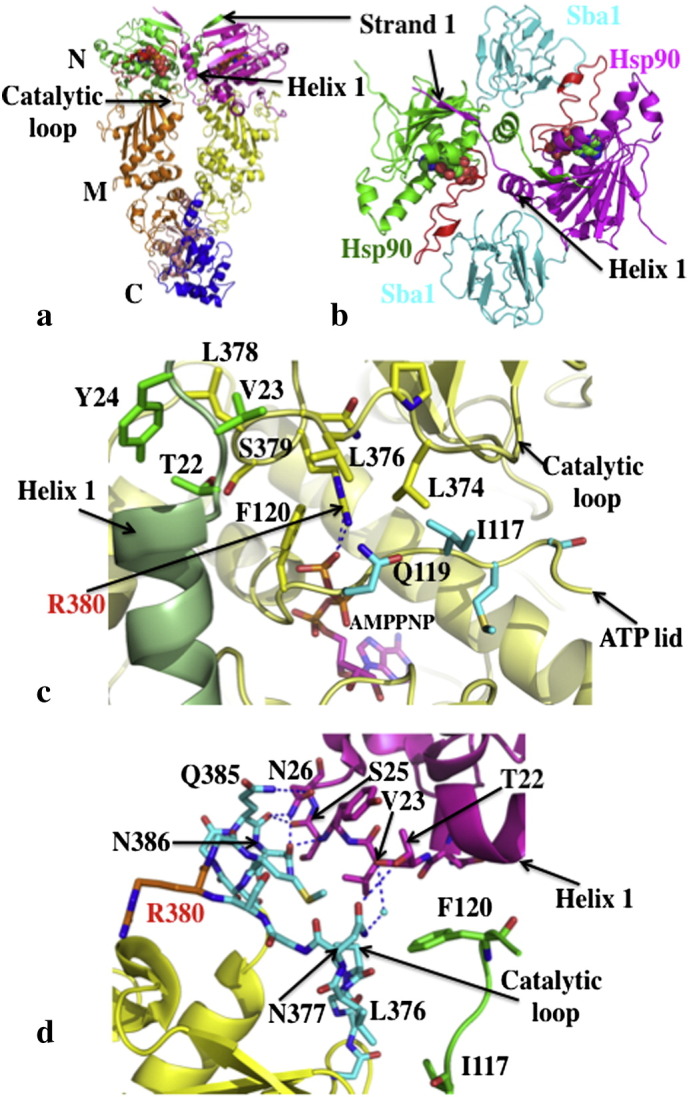
The catalytically active unit of Hsp90. a), Pymol cartoon of the Hsp90 dimer (pdb, 2CG9; Sba1 not shown). N-terminal domains (N) are in green and magenta, middle domains (M) in gold and yellow and the C-terminal domains (C) in salmon and blue. AMPPNP is shown as spheres bound to the N-terminal domains. b), Stabilization of the N-terminal domains of Hsp90 in their dimerized state by Sba1 (pdb, 2CG9). Sba1 is shown in cyan bound between the dimerised N-terminal domains of Hsp90 (green and magenta). The ATP lids (red) are in their closed conformation. α-helix 1 and β-strand 1, which undergo an exchange of position are indicated. AMPPNP is shown as spheres. c), Stabilization of the catalytic loop (yellow with yellow residues) by interaction between Thr 22 of the neighboring N-terminal domain (pale green with green residues) and Leu 378 of the catalytic loop and between Ile 117 of the ATP lid (yellow with cyan residues) and Leu 374 of the catalytic loop of the same monomer (pdb, 2CG9). Hydrogen bonds between the catalytic Arg 380 and AMPPNP are shown with dotted blue lines. d), Model of the domain interface between the N-terminal- and middle-domain of Hsp90 showing that the catalytic loop may remain in a closed inactive state and is thus not coupled to other structural changes resulting from the closed dimerized state of Hsp90. The middle domain (pdb, 1HK7; yellow) with a closed catalytic loop (cyan, except for Arg 380 which is shown in gold) was superimposed on the full-length closed Hsp90 structure (pdb, 2CG9; N-domain, magenta; middle domain, green). No steric clashes are observed and a number of hydrogen bonds can be formed, which are shown as dotted blue lines. Water molecules are shown as cyan spheres.

Understanding the mechanism of ATP hydrolysis is crucial to understanding the role that Hsp90 co-chaperones play and ultimately the mechanism of client protein activation. Direct evidence for this controversial activity [2–4] came from the crystal structure of the N-terminal domain of the *saccharomyces cerevisiae* Hsp90 in complex with ADP, which identified that the essential residues for ATP catalysis were conserved [5]. The focus then moved on to the precise mechanism by which ATP is hydrolyzed [6–9]. Support for the rate-limiting step being structural change [9], rather than ATP hydrolysis [6], emerged from the co-crystal structure between the middle domain of Hsp90 and the N-terminal domain of Aha1 [8]. Aha1 was seen to modulate the so-called catalytic loop of the middle domain of Hsp90 and therefore activate its ATPase activity. Categorical evidence that the formation of catalytically active units of Hsp90 involve a series of complex conformational changes [7] was seen with the full-length structure of Hsp90 in its closed N-terminally dimerized state, stabilized by Sba1 bound between its N-terminal domains [10] (Fig. 1a–b). This revealed the structural changes that form the catalytically active state involve a complicated process of conformational switches in different regions of the protein, which together conspire to assemble the active state [7,9–11]. It is now apparent that all Hsp90 homologs can hydrolyze ATP [12–15]. To understand the role of co-chaperones in Hsp90 complexes it is essential to understand the conformational cycle of Hsp90. Thus, I will give a brief outline of the ATP-coupled structural changes of Hsp90 before discussing the role of co-chaperones in Hsp90.

## 2 Structural changes and the catalytic unit of Hsp90

To achieve a closed catalytically active state, Hsp90 initially undergoes a structural rearrangement of its ATP lids, represented by residues Gly 94 to Gly 121 (*S. cerevisiae* numbering), which close over the bound ATP. The restructuring of this segment disrupts the packing of the ATP lid against α-helix 1 of the N-terminal domain. This in turn allows this helix and β-strand 1, residues 1 to 27, of each monomer of Hsp90 to remodel as part of an exchange between the N-terminal domains that occurs during dimerization (Fig. 1a–b). The repositioning of α-helix 1 is important as it sets up the dimerization interface for the N-terminal domains (Fig. 1b). Furthermore, the ATP lid in the closed state allows the catalytic loop residue (Arg 380) of the middle domain access to the ATP-binding site. The ATP lid in the closed position also helps to stabilize the catalytic loop in its open active state, by making a hydrophobic contact between its Ile 117 amino acid residue and Leu 374 of the catalytic loop (Fig. 1c). In addition, the repositioning of α-helix 1 allows its Thr 22 residue to interact with Leu 378 of the catalytic loop of the neighboring Hsp90 monomer and thereby stabilize it in an active state (Fig. 1c).

**Fig. 2:**
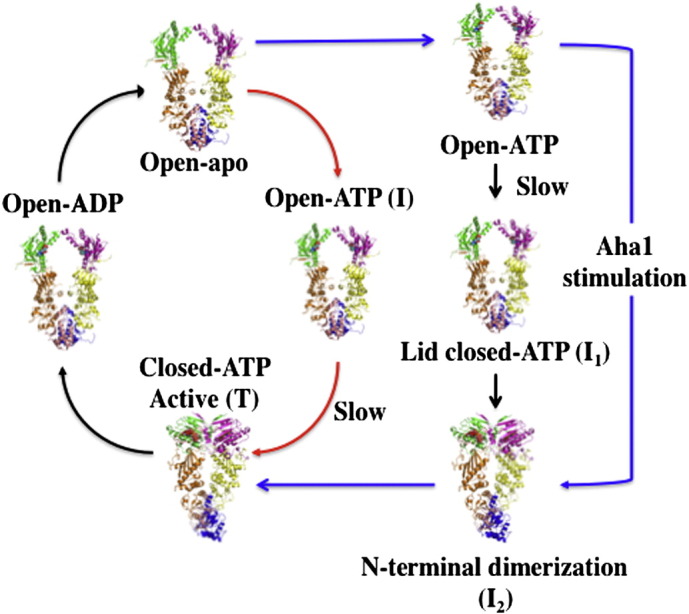
Kinetic cycles of the *S. cerevisiae* Hsp90 and *E. coli* Htpg. In the yeast cycle (blue and black arrows) the conformational changes leading to the catalytically active state (Closed-ATP-Active) involves transition via two intermediate conformations (I_1_ and I_2_). Aha1 is supposed to accelerate the cycle by bypassing the I_1_ sate. In the *E. coli* cycle (red and black arrows) a two-phase transition via an intermediate state (I) leads to the closed active (T) state. The slowest step in the cycles, both representing conformational change, are indicated. The open, closed and active state as well as the nucleotide state of the chaperone throughout the cycle is indicated.

Recent kinetic analysis [16,17] lends support to the rate-limiting step of ATP turnover being conformational change rather than ATP hydrolysis [6]. The first of these models [17] suggests that following binding of ATP to yeast Hsp90, the conformational changes leading to the catalytically active state involves a transition to two intermediate conformations (Fig. 2). The first, I_1_, results from ATP lid closure and release of the N-terminal segment of the N-terminal domain and has the lowest rate constant. I_2_ is then formed through dimerization of the N-domains. Finally, a third transition to the catalytically active state occurs by association of the N- and middle-domains and presumably interaction of ATP with the catalytic loop. The ATP is now trapped and committed to hydrolysis. In this model Aha1 bypasses the formation of the I_1_ state, thus presumably accelerating the rate-limiting step of the reaction. In another model, binding of ATP to the *Escherichia Coli* Hsp90 homolog, HtpG, leads to a two-phase transition, a rapid change to an intermediate state followed by a slower transition to the T (closed) state (Fig. 2). Interestingly, HtpG lacks an equivalent Aha1 homolog, which might account for this difference, assuming all states have been identified.

The above model suggests that Aha1 modulates specific early conformational switches that accelerate the rate-limiting step of the cycle? However, this might not be as simple as it appears at first sight. The activation of the Hsp90 ATPase activity by full-length Aha1 is significantly stronger than that by the N-terminal domain alone [15]. Thus, it is clear that Aha1 has additional affects on Hsp90 that have not as yet been structurally characterized. However, the N-terminal domain of Aha1 in complex with the middle domain of Hsp90 shows that Aha1 releases the catalytic loop of the middle domain, but this step seems to be, at least in the absence of Aha1, a late step conformational change. Furthermore, release of the catalytic loop might in itself influence earlier steps in the cycle, thus altering the kinetics. Therefore, it is possible that under different circumstances the precise details of the kinetics can vary due to changes in specific conformational switches of Hsp90 under different conditions. Currently, evidence suggests that Aha1 acts to modulate a number of different stages in the cycle. It could act directly to release the ATP lid either by displacing it or indirectly by modulation of the N-terminal sequence (β-strand 1 and α-helix 1) of the N-terminal domain that is intimately associated with the ATP lid. Alternatively, it might provide stability for the associating N-terminal domains during dimerization and or the association of the N- to middle-domains. Noteworthy, is the ability of Aha1 to suppress the F349A mutation, which is found in the N- and middle-domain interface, thus indicating that Aha1 might play a role in stabilizing the association of these domains. In fact, the binding of Aha1 on both the N- and middle-domains of Hsp90 [8,18] suggests that the domains of Hsp90 need to be closely associated for the complete Aha1–Hsp90 association to occur. It is also interesting to note that the association of the N- and middle-domains brings the N-terminal segments of the N-terminal domains close to the catalytic loops in their closed state. In fact, in the closed state the catalytic loops may potentially become hydrogen bonded to the N-terminal domains (Fig. 1d). Thus, the simplest function of the N-terminal domain of Aha1 appears to be as an activator of Hsp90 ATPase activity by destabilizing not only the interactions of the catalytic loop with the middle domain of Hsp90, but possibly also with the N-terminal domains of the chaperone.

The series of conformational switches or structural changes required to form the catalytically active state of Hsp90 makes it ideal for regulation by a variety of different co-chaperones. This is achieved by modulation of the individual switches of Hsp90. It also suggests that the conformational changes that occur in Hsp90 may not necessarily be coupled under all circumstances. This has been demonstrated in at least two different ways. Firstly, the activation of Hsp90 by full-length Aha1 and the N-terminal domain of Aha1 (or Hch1) do not stimulate the ATPase activity of Hsp90 to the same degree. Since full-length Aha1 stimulates Hsp90 ATPase far more so than the N-terminal domain alone, then it follows that other conformational changes required to achieve the fully active state might not be necessarily coupled to the movement of the catalytic loop. Secondly, Rar1 can activate Hsp90 in an open state. In this case it is assumed that the catalytic loop of Hsp90 is involved in the catalysis of ATP, but clearly not dependent on ATP lid closure and N-terminal dimerization. Such a system of conformational switches makes Hsp90 ideal for a variety of different regulatory mechanisms to operate and thus modulate the Hsp90 cycle in a tailored fashion that suits the maturation or activation of its client protein.

While the human and yeast Hsp90 systems appear to be similar there are some differences seen with the kinetics of the Trap1 and Grp94 orthologues. For Trap1 the rate-limiting step appears not to have yet been settled and is either hydrolysis or conformational change. However, whatever the situation commitment to hydrolysis appears to be absent [14]. Grp94 also lacks trapping of the bound ATP and commitment to hydrolysis [13]. These differences in the ATPase cycle may have evolved due to the differing requirements and co-chaperone associations and thus the specific regulation that Hsp90 is subject to.

## 3 Co-chaperone modulation of the catalytic unit

### 3.1 Sti1/Hop

**Fig. 3:**
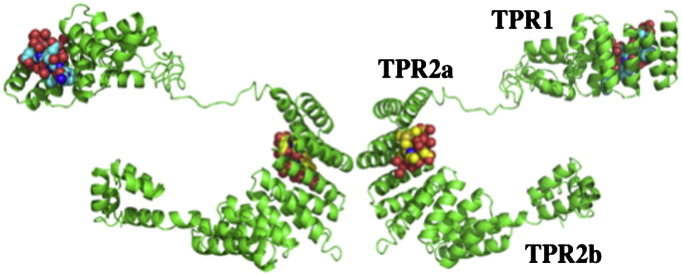
Model structure of Hop. Evidence suggests that although Hop/Sti1 may be dimeric in solution, in Hsp90 complexes Hop/Sti1 monomers may interact with Hsp90 independently. The TPR1 and TPR2a domains are shown bound with their Hsp70 and Hsp90 target peptides, respectively. Co-ordinates obtained as a kind gift from J. Guenter Grossmann.

The co-chaperone Sti1/Hop is responsible for the delivery of client proteins, such as steroid hormone receptors, to Hsp90 in a system that also involves the chaperone Hsp70. Regulation of the ATPase activity of Hsp90 was first shown with the potent inhibitor Sti1 [19]. The lack of a high resolution structure of Hop/Sti1 in complex with Hsp90 has held back our understanding of how this co-chaperone regulates the ATPase activity of Hsp90, although it is well established that the primary binding site is the MEEVD motif, at the extreme C-terminus, of Hsp90 [20]. Initial, biochemical and structural studies, with the yeast and human protein reveled additional contacts to the C-terminal-, middle- and N-terminal-domains of Hsp90 [19,21,22]. Using the structure of the individual domains a model was reconstructed that showed Hop to be a dimer, as previously suggested [19,23], and to have a butterfly like conformation (Fig. 3) [21]. The TPR2A domain was shown to be the main dimerization interface, which is consistent with previous work [19]. However, recent evidence has emerged that supports multiple interaction sites between Hop and Hsp90 [24] and suggests that for Hop each monomer can bind to Hsp90 independently [24]. Thus, it is conceivable that Hop can exist as a dimer, but its interaction with Hsp90 is as monomeric units [24] (Fig. 3). However, this study does confirm that Hop has multiple interaction sites with the C-terminal, middle- and N-terminal-domains of Hsp90, and thus supports previous studies showing that Sti1 is able to interact with the first 24 amino acid residues of the N-terminal domain and thus prevent its dimerization [22]. Furthermore, the formation of Hsp90 complexes bound with a single Hop monomer supports the finding that a single Hop molecule can inhibit the ATPase activity of Hsp90, while simultaneously allowing access of immunophillins and progression of the chaperone cycle [25]. Interestingly, the addition of AMPPNP to a Cpr6–Hsp90–Sti1 (cpr6 and Sti1 as monomers) complex resulted in a decrease in Sti1 binding in analytical ultracentrifugation experiments. This supports earlier work, which showed that the binding of Sti1 displaced geldanamycin from the N-terminal nucleotide-binding site of Hsp90 [19]. However, displacement of Sti1 was most pronounced in the presence of p23 [25].

The ability of Hop/Sti1 to inhibit the ATPase activity of Hsp90 might be required for client protein loading [19]. Hsp70 in its low affinity client-protein binding state, represented by its ATP state, would probably favor such a loading mechanism, and it would be logical to assume that this would be a prerequisite for transfer of the client protein from Hsp70 to Hsp90. However, the affect of Hop and Sti1 on the ATPase activity of Hsp70 is variable. Hop from rabbit reticulocytes has been shown to stimulate the ATPase activity of Hsp70 [26]. It has also been shown that Hop could lower the *K*_d_ value for the binding of ATP close to that of ADP and thus could act as a nucleotide exchange factor [26]. Similarly, the activation of the ATPase activity of Ssa1 (a yeast Hsp70) by Sti1 has been reported [27]. In contrast the human Hop protein appears not to be an effecter of the ATPase activity of Hsp70 and also does not act as a nucleotide exchange factor, but simply prefers to bind Hsp70 in its ADP state [27]. Whatever the effect of Hop or Sti1 on the ATPase activity of Hsp70, the release of the client protein must require that Hsp70 be converted to an ATP-like state so the client protein can be released. The molecular details of how this might be achieved are currently unknown.

### 3.2 Cdc37

**Fig. 4:**
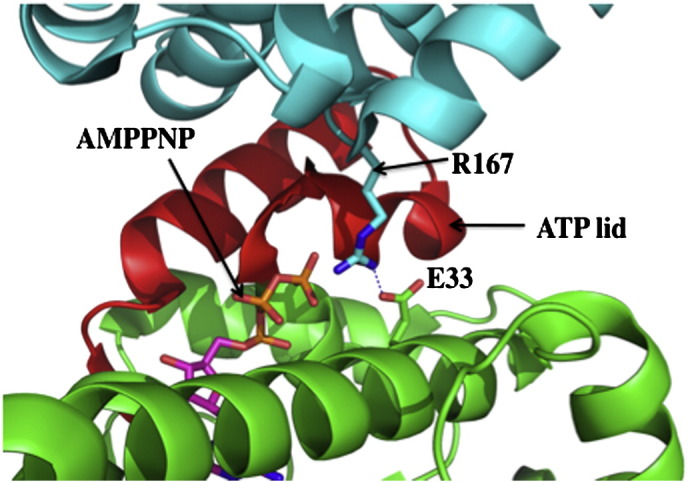
Inhibition of the ATPase cycle by Cdc37/p50. Cdc37/p50 is shown in cyan and the N-terminal domain of Hsp90 in green with a red ATP lid (pdb, 1US7). Arg 167 of Cdc37/p50 interacts with the catalytic Glu 33 and prevents it from hydrolyzing ATP. The non-hydrolysable analog of ATP, AMPPNP is shown in magenta. Hydrogen bonds are shown as dotted blue lines.

Another co-chaperone known to be involved in delivering client proteins to the Hsp90 complex is Cdc37/p50. Like Hop/Sti1, Cdc37 also inhibits the ATPase activity of Hsp90 [28]. In this case, however, the structural details for the molecular interactions between Cdc37/p50 and Hsp90 are well documented [29]. Human Cdc37 (p50) prevents N-terminal dimerization by binding between the N-terminal domains of Hsp90, interacting with the ATP lids themselves, thus physically blocking dimerization [29]. Furthermore, Arg 167 of Cdc37 hydrogen bonds with the catalytic Glu 33 residue of Hsp90 and prevents it from carrying out hydrolysis of ATP (Fig. 4) [29]. How exactly the client protein is transferred to Hsp90 and whether Cdc37/p50 alters the conformation of the kinase to achieve this is unknown.

### 3.3 Sgt1 and Rar1

**Fig. 5:**
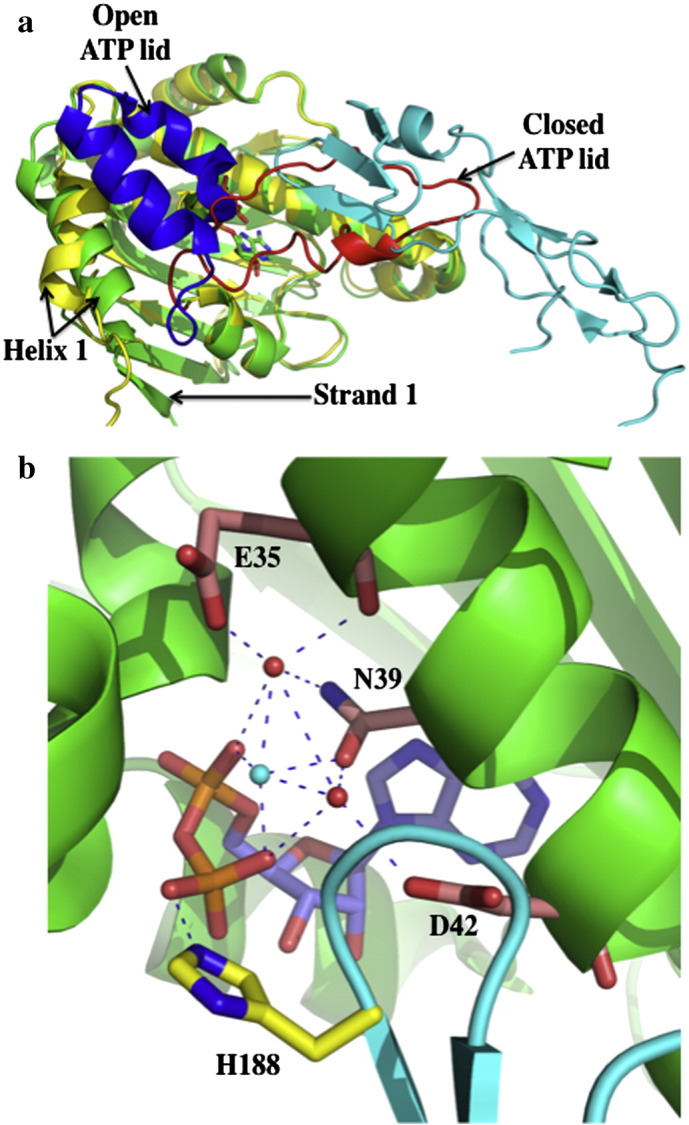
The stable Hsp90–Rar1 complex. a), Pymol cartoon showing the binding of the CHORD II domain of Rar1 (cyan) to the N-terminal domain of Hsp90 (pdb, 2XCM; open state, green; closed state, yellow ). The ATP lid in the open state is blue and in the closed state it is red. The closed state of the ATP lid is incompatible with the binding of the CHORD II domain. b), Pymol cartoon showing the interaction of the CHORD II domain of Rar1 with the ADP bound Hsp90 (pdb, 2XCM). The N-terminal domain of Hsp90 is shown in green with amino acids involved in binding ADP in salmon. CHORD II domain is shown in cyan with His 188 in yellow. ADP is shown in pale blue. Water molecules are shown as red spheres and the magnesium ion as a cyan sphere. Hydrogen bonds are shown as dotted blue lines.

Sgt1 is yet another co-chaperone that interacts with Hsp90 [30–32] and also appears to be involved in client protein loading through its ability to act as a hub for the formation of Hsp90 complexes [33–38]. Sgt1 and Rar1 play a central role in the innate immunity response in plants. Sgt1 has been found to associate with SCF E3 ubiquitin ligases, the CBF3 kinetochore complex, plant R proteins and related animal Nod-like receptors [39–46]. Sgt1 consists of three domains, an N-terminal TPR domain, a middle CS domain and a C-terminal SGS domain. The TPR domain is homologous to other TPR domains that bind the MEEVD motif of Hsp90. In this case, however, the TPR-domain of Sgt1 binds Skp1 [46], and its CS domain binds Hsp90 [46]. The molecular details of this interaction have been recently published [33]. The structure showed that the CS-domain is, as expected, homologous to Sba1/p23, but unlike Sba1/p23 it does not inhibit the ATPase activity of Hsp90 [33,46]. Consistent with this, the CS domain of Sgt1 does not bind directly to the ATP lids of Hsp90, as seen with Sba1. Thus, it does not inhibit the conformational cycle in the same way that Sba1 does. Instead, the CS domain of Sgt1 binds at a second site on the N-terminal domain of Hsp90. Interestingly although it does not affect ATPase activity of Hsp90 directly, it can recruit a second co-chaperone, Rar1 in plants, Chp1 and melusin in mammals, which in the case for Rar1 has been shown to be able to weakly stimulate the ATPase activity of Hsp90 [34]. Rar1 consists of two CHORD domains (CHORD I and II), of which each binds two zinc ions, whereas Chp1 and Melusin also contain a C-terminal CS domain. However, the structure of the N-terminal domain of Hsp90 in complex with the CS domain of Sgt1 and the CHORD II domain of Rar1 was recently published [34]. The CHORD II domain consists of two lobes forming a cylindrical structure (~ 54 Å long and ~ 13 Å in diameter). The C-terminal lobe consists of a three-stranded antiparallel β-sheet covered on one side by a short α-helix. The N-terminal lobe is devoid of secondary structure, apart from a short β-strand, which makes an antiparallel interaction with a β-strand extending from the C-terminal lobe, crossing back onto the N-terminal lobe. Uniquely, the stimulation of the ATPase activity by the CHORD II domain of Rar1 occurs while Hsp90 is in an open conformation, resulting in a stable ADP bound open state complex (Fig. 5a–b). The structure of this complex revealed that the CHORD II domain might replace the ATP lid and simultaneously modulate the catalytic loop of the middle domain to achieve an activation of Hsp90. The Rar1 (CHORD II)–Hsp90–Sgt1 (CS domain) complex was also shown to be most stable in an Hsp90-ADP state [34]. Thus, the Rar1 stimulated hydrolysis of ATP by Hsp90 would convert a Rar1–Hsp90–Sgt1 complex in to a stable ADP-bound complex. As these complexes are involved in the innate immunity response, in both plants and animals, it is interesting to note that a stable complex could act as a long lived sensor for small molecules from invasive organisms so that the host could act immediately, without the delay of initiating transcription in the first instance. Thus, in this case client loading seems to occur onto a stable inactive Hsp90 complex brought about by the action of Sgt1 and Rar1.

### 3.4 Sba1/p23

**Fig. 6:**
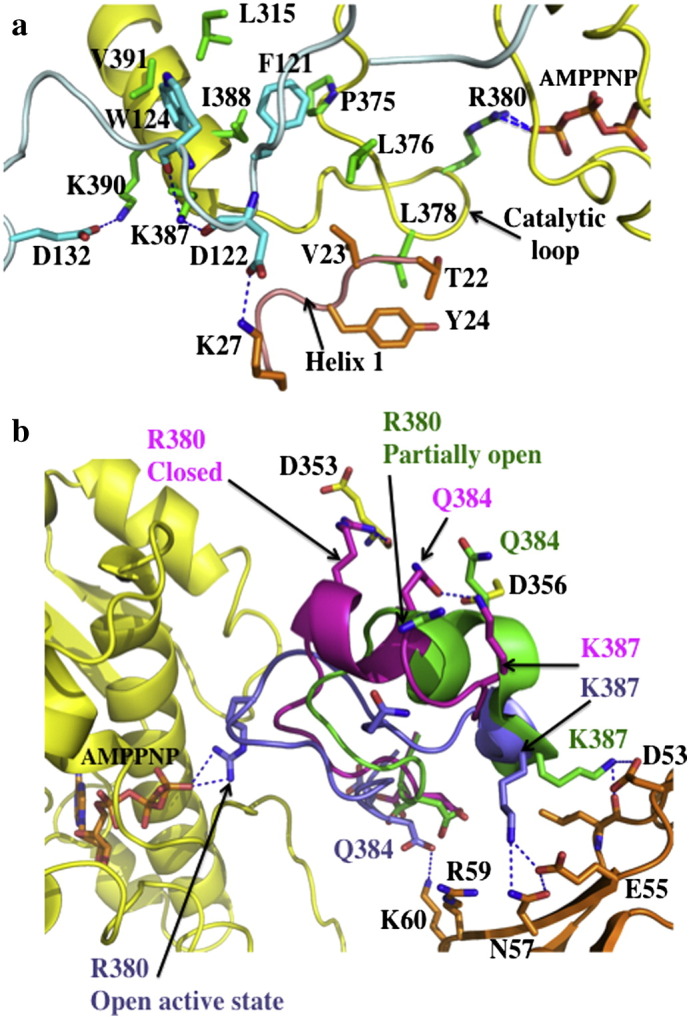
ATPase modulated states of Hsp90. a), The ‘inhibited’ state of Sba1 bound Hsp90 (pdb, 2CG9). Sba1 is shown in light blue with amino acid residues in cyan. The middle domain of Hsp90 is shown in yellow with amino acids residues in green. The N-terminal domain sequences from the neighboring Hsp90 monomer are shown in gold. ATP is shown bound to Arg 380 by hydrogen bounds (dotted blue lines). Trp 124 of Sba1 binds into a hydrophobic pocket formed by the Hsp90 residues, Leu 315, Ile 388 and Val 391, which signals the catalytic loop to be released into its active open conformation. This state represents an ‘inhibited’ form of Hsp90 that is committed to hydrolyzing ATP. b), Modulation of the catalytic loop of Hsp90. Hsp90 is shown in yellow (pdb2CG9; middle- and C-terminal-domain not shown). The catalytic loop is shown in magenta (closed state; pdb, 1HK7), green (partially open state of the N-domain Aha1 — middle domain of Hsp90 complex, pdb 1USU) and the open active state (blue) of the Sba1-bound structure (pdb 2CG9). Aha1 is in gold. The partially open state of the catalytic loop in the N-Aha1 — middle-Hsp90 complex results because in this structure Arg 380 cannot interact with ATP. Hydrogen bonds are shown as dotted blue lines.

Unlike the above co-chaperones discussed so far, Sba1/p23 is not involved in delivering client proteins to Hsp90. Instead it appears to act to stabilize such complexes and the best example of this type of complex is that of steroid hormone receptors. While Sba1/p23 can bind Hsp90 in an apo state these co-chaperones show higher affinity for the ATP bound state of Hsp90 and therefore for its closed N-terminally dimerized state [7,47]. The mechanism that Sba1 uses to inhibit the ATPase activity of Hsp90 is somewhat different to that of Hop and Cdc37. Unlike Sti1, Cdc37 and Sgt1, Sba1/p23 acts later on in the ATPase cycle of Hsp90, which it appears to slow down. Initially, it was puzzling as to why yeast Sba1 only partially inhibited (slowed) Hsp90 ATPase activity [15]. A more robust inhibition was, however, seen with p23, the human homolog of Sba1 [47]. The structure of the full-length yeast Hsp90 in complex with Sba1 and AMPPNP helped to explain this observation (Fig. 6a) [10]. The structure showed that while Sba1 binds to the closed N-terminally dimerised Hsp90, locking the N-terminal domains together, it also simultaneously causes the release and stabilization of the middle domain catalytic loop in its active conformation. Consequently, the complex is committed at some point to hydrolyzing the bound ATP. Upon hydrolysis the complex would release Sba1, whose binding is dependent on the closed N-terminally dimerized state of Hsp90. Thus, Sba1 rather than halting the chaperone cycle, it uniquely slows it down, perhaps regulating the time a particular client is held in a specific conformation [10].

Recently, the structure of the CS domain of a variety of NudC and NudC-like proteins has been published [48] or deposited in the pdb database (*Encephalitozoon Cuniculi* NudC, human NudCL and NudCL2; pdb ID 2O30, 1WGV and 2RH0, respectively). Like Sba1 and p23, the human NudC has also been shown to be an inhibitor of the ATPase activity of Hsp90. However, the precise molecular details have not yet been established.

### 3.5 The activator of Hsp90 ATPase, Aha1

The co-chaperone Aha1, like Sba1/p23, is not involved in client protein loading. However, unlike Sba1/p23 it acts to accelerate the ATPase cycle of Hsp90 and presumably the activation and maturation of client proteins. Aha1 remains the most potent co-chaperone activator of the ATPase activity of Hsp90 found to date [8,15]. The N-terminal domain of Aha1 is known to be able to modulate the catalytic loop of the middle domain of Hsp90, stabilizing it in an open active-state. Full-length Aha1 is a significantly more potent activator of the ATPase activity. However, how it achieves this is yet to be determined. Recently, it was shown that the C-terminal domain of Aha1 binds to the dimerized N-terminal domains of Hsp90 [18]. It is conceivable that here again we have another mechanism by which the ATPase activity of Hsp90 can be modulated. It is possible that the C-terminal domain of Aha1 might either initiate N-terminal dimerization or may help to stabilize the N-terminally dimerized state or the association of the N- and middle-domains of Hsp90 (Fig. 6b). However, further structural and biochemical work is required to categorically show the exact mechanism.

### 3.6 Tah1 and Pih1

Another co-chaperone that has been shown to activate the ATPase activity of Hsp90 is Tah1. Tah1, together with Pih1 form part of the chromosome remodeling complexes, Ino80 and SWR-C by interacting with Rvb1 and Rvb2 [49], as well as interacting with RNA processing factors, Rrp43p and Nop58p [50]. Activation by Tah1 of the Hsp90 ATPase activity is very weak [51], but has been confirmed by another study [52]. However, as a complex with Pih1, which binds to both Tah1 and Hsp90, it inhibits the ATPase activity of Hsp90 [52]. Interestingly, this again appears to be a mechanism by which the ATPase activity of Hsp90 is halted, thus allowing client protein loading and complex formation.

### 3.7 Immunophilins

Cpr6, an immunophilin co-chaperone of Hsp90, has been shown to weakly stimulate the ATPase activity of Hsp90 [15]. However, another group has not yet confirmed this activation and thus the details of how this might occur and its significance remain unknown. In contrast, FKBP59 failed to stimulate the ATPase activity of Hsp90, although it could stimulate the GR ligand-binding domain activated activity of Hsp90 [47]. These results raise the important question of whether other immunophilins (for example AIP/XAP2/ARA9) also regulate Hsp90 ATPase activity and what purpose such activation serves? The role of immunophilins in Hsp90 complexes is currently held back by the fact that there are currently no known structures of immunophilin complexes with Hsp90 as far as I am aware. However, it is clear that immunophilins may not play a client-loading role in all cases. In particular their incorporation into steroid hormone receptors occurs after steroid hormone receptor loading onto Hsp90 [53,54].

### 3.8 Client protein activation

The first report showing that activation of the weak ATPase activity of Hsp90 was with the client protein ligand-binding domain of glucocorticoid receptor [55]. However, in this study the ligand-binding domain was found to be dimeric rather than monomeric. Untransformed glucocorticoid receptor is found in cytosolic preparations as an 8–9 sv *(M*_r_*,* 300,000) heteromeric complex consisting of a single molecule of the receptor [56,57] and two molecules [58] of a non-steroid-binding phosphoprotein, Hsp90 [59–66]. However, whether ATPase activation of Hsp90 is a common feature for all client proteins has not been established and it also raises the question of whether Sba1 would be able to stabilize complexes, which are actively hydrolyzing ATP and thus promoting an open state conformation. For kinase complexes the presence of Cdc37/p50 would promote an open inactive state [28]. Whether, there are differences in the regulation of Hsp90 ATPase activity by different client proteins remains to be seen.

## 4 C-terminal domain ‘ATP binding site’

**Fig. 7:**
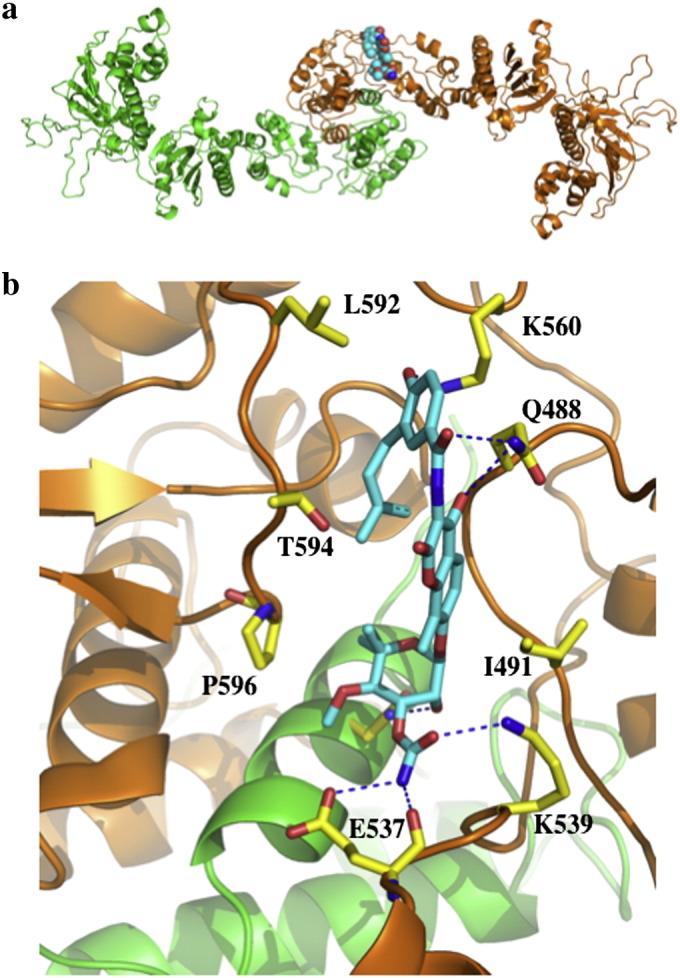
Model of the C-terminal small molecule-binding site of Hsp90. a), Pymol cartoon showing the proposed site of interaction of novobiocin (cyan spheres) with the Hsp90 dimer (green and gold; co-ordinates were a kind gift from Brian S. J. Blagg. b), Proposed interactions of novobiocin (cyan) with the C-terminal domains of Hsp90. Novobiocin is bound close to the C-terminal dimerization interface of Hsp90 and makes contact with both monomers of Hsp90 (green and gold). Hydrogen bonds are shown as dotted blue lines.

Clearly the ATPase activity of Hsp90 is important for the activation of client proteins. Although, much of this process as we understand it is due to the hydrolysis of ATP at the N-terminal domains of Hsp90, a C-terminal ATP-binding site has also been reported [67–71]. However, elucidation of the full-length structures of yeast Hsp90 [10], canine Grp94 [72] and *E. coli* HtpG [73] do not obviously show an ATP-binding site *per se.* The development of C-terminal inhibitors of Hsp90 suitable for the clinic has been hampered by the lack of a crystallographic structure of a small molecule bound to the C-terminal domain of Hsp90. There is, in fact, much interest in achieving this as such inhibitors elicit unique effects on Hsp90's conformation, ATPase activity and its interactions with co-chaperones and client proteins [69,70,74–82]. Recently a C-terminal binding site has been elucidated and a structure bound with novobiocin modeled (Fig. 7a–b) [83]. Although this site appears not to be an ATP-binding pocket with catalytic activity, occupancy of this site by a small molecule prevents ATP-binding to the N-terminal domain [70]. How this is achieved is not yet known, but might involve co-operativity between the N- and C-terminal domains of Hsp90 [84]. However, it is clear that the binding of a small molecule to this site would disrupt the ability of Hsp90 to undergo N-terminal dimerization. It might also prevent C-terminal dissociation of the C-terminal dimerization domains [84]. This would not only inhibit the ATPase conformational cycle of Hsp90, it would also disrupt the interaction of specific co-chaperones and client proteins with Hsp90.

## 5 Post-translation modifications affecting the ATPase activity

### 5.1 Phosphorylation

**Fig. 8:**
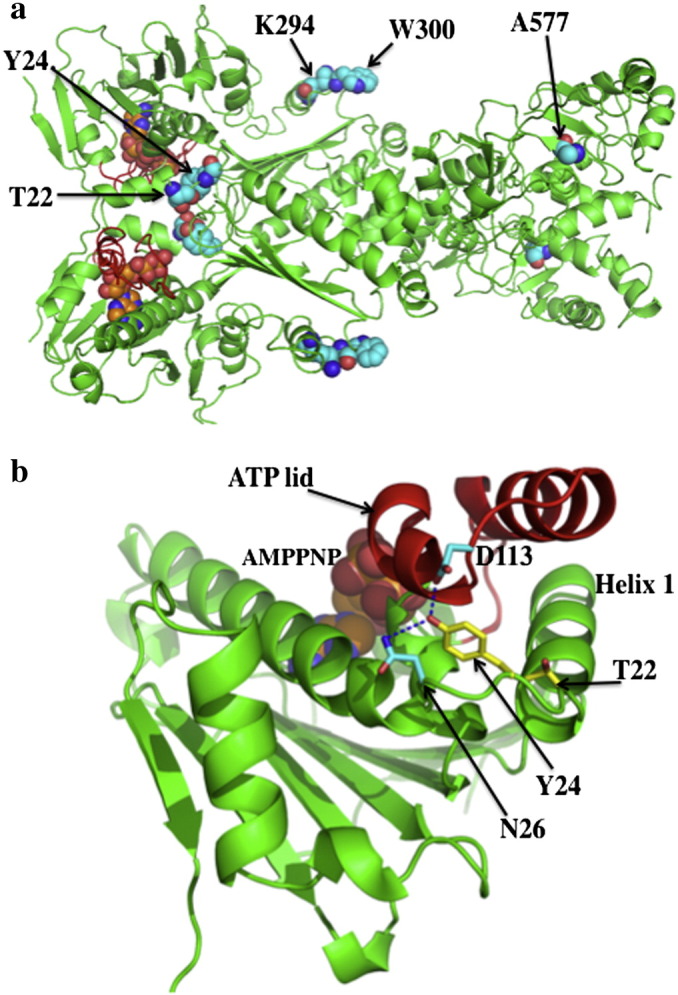
Sites of post-translational modification of Hsp90. a), The closed active conformation of yeast Hsp90 showing amino acid residues that are phosphorylated (Thr 22 and Tyr 24), acetylated (K294), nitrosylated (Cys 598 In Hsp90α; equivalent position in yeast Hsp90 is Ala 577) and involved in client protein binding (Trp 300). ATP and selected amino acid residues are shown as gold and cyan spheres, respectively. b), Position of amino acid residues (Thr 22 and Tyr 24, yellow) in the open state of the N-terminal domain of yeast Hsp90. ATP is shown in spheres. Hydrogen bounds are shown as dotted blue lines.

The topic of post-translational modifications is treated comprehensively by Len Neckers and Mehdi Mollapour in this special edition on Hsp90 in the chapter titled “Post-translational modifications of Hsp90 and their contributions to chaperone regulation”. Consequently, I will briefly discuss the main aspects that affect the ATPase activity of Hsp90 and complex formation. A number of Hsp90 post-translational modifications have been detected that affect its ATPase activity. Tyr 24 in yeast- and Tyr 38 in human Hsp90 have been shown to be a common site upon which Hsp90 is phosphorylated [85] (Fig. 8a–b). In yeast the tyrosine kinase responsible for this phosphorylation was identified as Swe1^Wee1^ [85]. Mutation of Tyr 24 to a non-phosphorylatable phenylalanine residue had no affect on its ATPase activity. However, mutation to a glutamate, a potential mimetic of the phosphorylated state, almost completely abolished Hsp90 ATPase activity. *In vivo*, as sole Hsp90, Y24E and Y24D mutants could not support viability due to their low ATPase activity. However, using the nonphosphorylatable mutants, Y24F and Y38F, indicated that phosphorylation at this site is important for productive chaperoning of kinases and for suppressing heat shock factor activity, but had minimal affect on the chaperoning of steroid hormone receptor client proteins. Thus, a mechanism by which Hsp90 could be targeted to specific groups of client proteins could operate by modulation of the phosphorylated state of Tyr 24 in yeast and Tyr 38 in human cells.

Another phosphorylation site that has been identified is Thr 22 in yeast and Thr 36 in human Hsp90 [86]. The phosphorylation of Thr 22 and Thr 36 was shown to be dependent on casein kinase II activity. Like Tyr 24, Thr 22 forms part of an interacting group of residues that come together to form important interactions with the catalytic-loop residue Leu 378, from the neighboring monomer of Hsp90 (Figs. 1c and 6a). A previously described mutation T22I, originally isolated as a temperature sensitive mutation in yeast [87], was shown to increase the ATPase activity of Hsp90 [9]. The increased hydrophobicity of the T22I mutation and its interaction with the catalytic-loop residue Leu 378, are consistent with this mutation stimulating the ATPase activity of Hsp90. Mutation of Thr 22 to alanine does not affect the ATPase activity of Hsp90, however, mutation to glutamate, a possible phosphomimetic mutation, resulted in a decrease in the ATPase activity of Hsp90 [86]. As with the Y24E mutation, the T22E mutant showed differential effects on the chaperoning of specific client proteins. Thus, a negative affect was seen on the chaperoning of v-Src and Ste11, whereas glucocorticoid receptor (GR) activation was increased by approximately four-fold and the stability of the cystic fibrosis transmembrane regulator (CFTR) was improved. In the case for the human mutation, T36E, loss of interaction with v-Src, Raf1, ErbB2, Cdk4 and GR was observed, whereas CFTR protein levels increased.

Changes in the association of client proteins with phosphorylated Hsp90 might occur due to direct effects on their association or indirectly due to effects on co-chaperone interaction with Hsp90. With Y24F and wild type Hsp90 in a *Swe1Δ* background the association of Aha1 was completely abolished, sba1 interaction reduced, while Cdc37/p50 and Sti1/Hop association was unaffected relative to wild type Hsp90. A similar situation was found using the human Y38F mutant. Effects on co-chaperone interactions with the T22A and T22E mutants were also seen. The association of Aha1 was completely abolished, while that of Cdc37 was reduced. Interactions with Sti1 and Sba1 were unaffected. Similar results were seen with the human T38A and T38E mutants. Aha1 association was completely diminished, while that of p50 was mainly reduced with the T38E mutant. Hop interaction was again unaffected, but p23 interaction was slightly reduced. Thus, these results suggest that changes in the phosphorylation state of Hsp90 could target the chaperone to distinct types of complexes.

The list of Hsp90 phosphorylation sites identified is now fairly extensive (see [88]) and is probably beyond the scope of this review. However, the examples above raise the possibility that phosphorylation of Hsp90 acts to modulate the conformational cycle of Hsp90 and thus mold the chaperone cycle to specific client proteins. This is an interesting point as the clientele of Hsp90 are structurally very varied and would have specific requirements for their maturation and activation. However, changes in the chaperone cycle of Hsp90 could result by a number of mechanism including direct changes in the ATPase activity of Hsp90, changes in its interaction with co-chaperones or its clientele.

### 5.2 Acetylation

Hsp90 acetylation has also been reported [89–92], however, affects on the ATPase activity of Hsp90 have not been determined. A direct functional analysis at one specific site, Lys 294 of yeast Hsp90, has been carried out [91] (Fig. 8a). Mutations, mimicking acetylation at this site, K294Q and K294A, drastically reduced interaction with ErbB2, V-Src, mutant p53 and HIF1α, suggesting a universal impact on Hsp90 interaction with client proteins. Interestingly, Lys 294 lies close to, and on the same face, as Trp 300, which was previously identified as a client protein-binding site in the middle domain of Hsp90 (Fig. 8a) [93]. Mutation at this site did not affect Hsp90 ATPase activity, but caused severe defects in growth and v-Src activation at 30 °C and was lethal at 37 °C. Comparing co-chaperone associations between the conservative mutation, K294R, and the acetylation mimetic mutants, K294Q and K294A, showed that interactions were altered. Interactions with p23, p50, p60, Hsp70 and CHIP were all reduced with K294A and K294Q, except that the p60 association with K294Q was only slightly affected, relative to wild type Hsp90. Association of Aha1 and FKBP52 was completely abolished with K294A and K294Q, while the K294R mutant showed increased interactions. Clearly, as with phosphorylation effects on client protein associations might be either direct or indirect due to changes in co-chaperone associations with Hsp90. However, it is clear that acetylation plays an important role in regulating Hsp90 complex formation and perhaps at a universal level.

### 5.3 S-nitrosylation

S-nitrosylation of Cys 598 is another post-translational modification that has been observed for human Hsp90α [94,95]. S-nitrosylation of Hsp90α results in the inhibition of its ATPase activity and a decrease in the ability to activate eNOS [95]. A similar inhibition of activity has been seen in the nitrosylated mutant, A577C, of the yeast Hsp90 protein (Fig. 8a) [94]. Mutation of Ala 577 to either isoleucine or asparigine appears to alter the dimerization of the C-terminal and N-terminal domains and consequently would affect client protein activation and co-chaperone association, but as far as I am aware this has not been studied in detail.

## 6 Mechanisms of drug resistance compatible with Hsp90 ATPase activity

Hsp90 has been the subject of intense research to develop potent inhibitors that are effective against cancer and a number of drugs have entered clinical trials (recently reviewed in [96]). The conservation of the Hsp90 N-terminal domain residues involved in ATP binding and hydrolysis is high. The ATPase activity of Hsp90 is also essential for the viability of cells [97]. Together, this suggests that mutations of residues involved in the ATPase activity of Hsp90 that lead to drug resistance are unlikely. So far resistance to 17-AAG has been seen in cells with low NAD9P0H/quinone oxidoreductase I (NQ01) activity [98–100]. NQO1 affects the degree of intracellular reduction of these inhibitors to the hydroquinone, a reduced state in which they are more potent inhibitors of Hsp90 [100]. Resistance is also seen by overexpression of P-glycoprotein and/or MRP-1 [101,102]. However, resistance due to point mutations of the active site of Hsp90 has so far not emerged from the clinic.

**Fig. 9:**
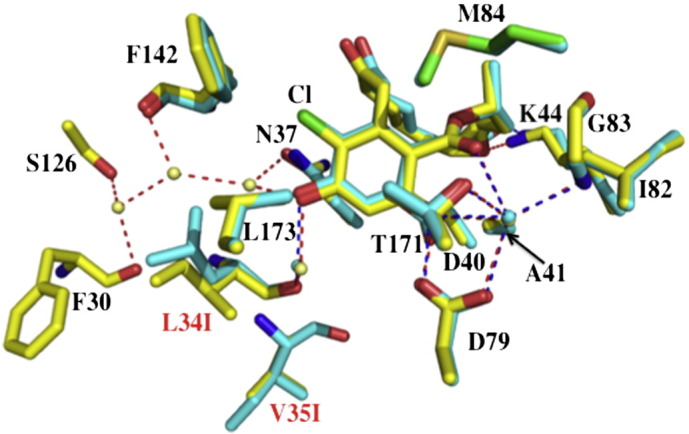
Radicicol resistance by modification of the ATP-binding site of Hsp90. A single mutation, L34I results in the opening of a pocket that alters the water structure of the active site. Three additional water molecules enter and repel the chlorine (Cl) of radicicol thus decreasing its affinity for Hsp90. Water molecules are shown as pale yellow (L34I) and cyan (wild type) spheres. Radicicol bound to the mutant protein is shown in yellow while that bound to wild type Hsp90 is in cyan. Amino acid residues of L34I are in yellow and residues of the wild type protein are in cyan. Mutations are shown as L34I and V35I in red text. Dotted red (L34I) and blue lines (wild type) represent hydrogen bonds.

For radicicol a single point mutation has been identified, L34I (yeast Hsp90) that leads to a partial resistance against radicicol binding, while preserving the binding of ATP and the ATPase activity of the protein. Thus, in all probability interactions with client proteins and co-chaperones are likely to be unaffected, although this was not tested. The molecular basis for resistance is due to changes in the water structure of the ATP-binding domain that repel the chlorine atom of radicicol (Fig. 9). The L34I mutation showed no cross-resistance to geldanamycin. Other point mutations that lead to resistance include Hsp90α I123T and A121N and Hsp90β I128T, A116N and T31I [103,104]. It appears that the increased resistance seen with such mutants might be due to enhanced interactions with Aha1 [103]. In fact, depleting the levels of Hsp90 co-chaperones such as Aha1, Cdc37 and p23 have been shown to increase resistance to Hsp90 inhibition in cells [105–107].

Like radicicol, geldanamycin is also a naturally produced antibiotic. Consequently it is highly likely that mechanisms involving the alteration of the ATP-binding site of Hsp90 exist. However, the lack of any mutations seen in the clinic, is not only encouraging from a clinical point of view, but suggests that these changes are likely to be complex in nature (see note as supporting evidence).

## 7 Concluding remarks

The conformational changes in Hsp90 are central to its role as a chaperone and co-chaperones play an important role in regulating them. Initiated by the binding of ATP these structural changes are subject to a whole host of regulatory mechanisms. The question of whether these changes are coupled or not, depends on the situation and the precise layer of regulation at that time. The current evidence suggests that the co-chaperones play a number of different roles in Hsp90. To date, co-chaperones have been identified that are responsible for the loading of client protein. These include Hop/Sti1, Cdc37/p50, Tah1–Pih1 complex and Sgt1–Rar1 complex. Others activate the ATPase activity of Hsp90. The most potent of these is Aha1, and it is presumed that Aha1 acts to increase the rate of activation and maturation of client proteins. Finally, we see that Sba1 acts to slow and stabilize client protein complexes and here we assume that the client might be stabilized in a particular conformation in an activatable state. The complex set of structural changes required to bring about a catalytically active state of Hsp90 has allowed a cohort of different regulatory co-chaperone mechanisms to act on Hsp90. However, the ‘great unknown’ that remains in understanding the Hsp90 chaperone cycle is what the conformational changes of Hsp90 do to activate or cause the maturation of client proteins. This major question needs to be addressed to further advance our understanding of the Hsp90 chaperone cycle.
